# The effect of surface charge on photocatalytic degradation of methylene blue dye using chargeable titania nanoparticles

**DOI:** 10.1038/s41598-018-25673-5

**Published:** 2018-05-08

**Authors:** Fadhel Azeez, Entesar Al-Hetlani, Mona Arafa, Yasser Abdelmonem, Ahmed Abdel Nazeer, Mohamed O. Amin, Metwally Madkour

**Affiliations:** 1 Chemical Engineering Department, Faculty of Engineering and petroleum, Kuwait University, P.O. Box 5969, Safat, 13060 Kuwait; 2 Chemistry Department, Faculty οf Science, Kuwait University, P.Ο. Bοx 5969, Safat, 13060 Kuwait; 3 Chemistry Department, Faculty οf Science, Menoufia University, 32511 Shebin El-Kοm, Egypt; 4 Electrochemistry Laboratory, Physical Chemistry Department, National Research Centre, Dokki P.O. 12622, Giza, Egypt

## Abstract

Herein, a simple approach based on tailoring the surface charge of nanoparticles, NPs, during the preparation to boost the electrostatic attraction between NPs and the organic pollutant was investigated. In this study, chargeable titania nanoparticles (TiΟ_2_ NPs) were synthesized via a hydrothermal route under different pH conditions (pH = 1.6, 7.0 and 10). The prepared TiΟ_2_ NPs were fully characterized via various techniques including; transmission electron microscopy (TEM), X-ray diffraction (XRD), N_2_ adsorption/desorption, X-ray photoelectron spectroscopy (XPS), Ultraviolet–visible spectroscopy (UV-Vis) and dynamic light scattering (DLS). The influence of the preparation pH on the particle size, surface area and band gap was investigated and showed pH-dependent behavior. The results revealed that upon increasing the pH value, the particle size decreases and lead to larger surface area with less particles agglomeration. Additionally, the effect of pH on the surface charge was monitored by XPS to determine the amount of hydroxyl groups on the TiO_2_ NPs surface. Furthermore, the photocatalytic activity of the prepared TiΟ_2_ NPs towards methylene blue (MB) photodegradation was manifested. The variation in the preparation pH affected the point of zero charge (pH_PZC_) of TiO_2_ NPs, subsequently, different photocatalytic activities based on electrostatic interactions were observed. The optimum efficiency obtained was 97% at a degradation rate of 0.018 min^−1^ using TiO_2_ NPs prepared at pH 10.

## Introduction

One of the sources of water pollution is the wastewater generated from textile-plants using different dyestuffs^1,2^. Different chemical and biological changes for dyes can occur which consume the dissolved oxygen in the water bodies. Moreover, dyes have high toxicity which endangers aquatic life^3,4^. The different traditional methods used for the treatment of pollutants textile dyes in water include various physical, biological, and chemical processes. Different advanced treatment techniques combining physical and chemical principles based on adsorption, ultrasonic decomposition, electrocoagulation, advanced chemical oxidation (ACO), chemical coagulation, nano filtration and sedimentation were employed. Most of these treatment methods have some deficiencies including high energy-waste, high cost, and production of secondary pollutants during the process of treatment^5,6^.

Exploring properties of nanomaterials for different applications is an attractive research area. One of the commonly used nanomaterials is titania nanoparticles (TiO_2_ NPs) due to their unique characteristics including optical, electronic and catalytic properties, which make them ideal candidates for different industrial applications such as photovoltaics, fillers, catalyst supports, and photocatalysis of wastewater treatment^2,4,6–8^.

Therefore, the photocatalytic process based on TiO_2_ semiconductor under UV light illumination showed great advantages in wastewater pollutants treatment over the traditional techniques. It is considered important and promising for the treatment of organic waste in the atmospheric and aquatic environments. However, there are two major shortcomings that impede the industrial applicability of TiO_2_ photocatalysis which are: 1) the large band gap which requires an ultraviolet illumination^9^ and 2) the high electron–hole pair recombination rate^10^. Thus, many efforts have been devoted to surmount the issues associated with the TiO_2_ NPs^11^.

Therefore, many studies have been published in attempt to overcome such limitations. An alternative approach is to design novel catalysts that exhibit high activity when illuminated by UV or visible light and have low recombination rates. For example, doping TiO_2_ NPs with noble metals^12^, transition metals^13^, metalloids^14^ or anions^15^ is widely utilized approach to minimize the band gap energy and recombination rate. Another approach which was extensively explored to enhance the photocatalytic activity is coupling different semiconductor particles with TiO_2_ NPs such as TiO_2_-CdS^16^, TiO_2_-WO_3_^17^ and TiO_2_-SnO_2_^18^. Coupling reduces the recombination rate and increases the energy range of the photoexcitation. Hybridization with conjugated materials has been also investigated, to improve the transportation of the photocarriers during photocatalysis due to their excellent electronic properties. For instance, Zhang *et al*. utilized graphite layers and C60 to enhance the photocatalytic activity of TiO_2_ NPs^19,20^. An alternative approach to prepare an efficient photocatalyst by fabricating core-shell geometry using two semiconductors was explored to enhance the emissive properties of the photocatalyst hence its photocatalytic activity^21^.

Generally, the pH of the solution is a key factor that affects directly the photoeffficiency of photocatalysts. This is due to the fact that the pH governs the surface characteristics and the size of aggregated nanoparticles, along with the charge of organic molecules, and finally governs the adsorption capacity of molecules onto nanoparticles surface and the concentration of hydroxyl reactive radicals^22^. Many attempts were devoted to better exploitation of surface charge properties to achieve superior photo reactivity such as such as surface doping and sensitization, creation of surface heterojunctions, modification with co‐catalysts, increase in the accessible surface areas, and usage of surface F effects and exposure of highly reactive facets^23,24^. However, all of these approaches are considered to be laborious, costly and require meticulous design.

Therefore, we propose an alternative and simple approach based on adjusting the pH value of TiO_2_ NPs to boost the electrostatic attraction between the surface of NPs and the dye hence improving the photodegradation efficiency. Initially, TiO_2_ NPs were prepared using different ethanol: water ratios and calcination temperatures. Yet, the ratio of ethanol: water did not play a major role in the properties of the formed TiO_2_ NPs. Considering the effect of calcination temperatures, as the calcination temperature increased a triple phase mix (rutile, brookite and anatase) was obtained. Then, the optimum TiO_2_ NPs were further prepared at different pH values and their point of zero charge (pH_PZC_) were measured and correlated with their photocatalytic efficiencies.

## Experimental

### Preparation of TiO_2_ NPs

TiO_2_ NPs were prepared by sol-gel method via hydrolysis of titanium tetrachloride (TiCl_4_). In a typical procedure, 4 mL of TiCl_4_ was added dropwise into a mixture of ethanol and distilled water with different ratios (1:1, 4:1 and 3:2 *v/v*). The mixture was refluxed at 80 °C under continuous stirring until a white suspension of TiO_2_ NPs was formed after approximately 4 hours. The NPs were precipitated by centrifugation at 5300 rpm for 30 minutes. The precipitate was filtered and washed several times and was dried overnight at 50 °C.

### Hydrothermal modification of TiO_2_ NPs

Hydrothermally modified TiO_2_ NPs were synthesized according to the same procedure in section 2.1 with some modifications using a mixture of ethanol and distilled water (4:1 *v/v*). The formed NPs were transferred into a 300 mL Teflon lined stainless steel autoclave at 150 °C for 5 hours. The suspension was then centrifuged at 5300 rpm for 30 minutes. The resultant NPs were dried overnight at 50 °C.

Furthermore, the synthesized TiO_2_ NPs were calcined at different temperatures: 100 °C, 200 °C, 300 °C, 400 °C and 500 °C. Finally, the effect of pH on the synthesized NPs was investigated at different pH values: 1.6, 7.0 and 10 by adjusting the pH of the ethanol: water ratio prior to introducing TiCl_4_.

### TiO_2_ NPs Characterization

Surface and bulk characteristics of the TiO_2_ NPs were evaluated using X-ray powder diffraction (XRD) patterns, obtained using Bruker, D8 ADVANCE diffractometer with Cu K_α_ (*λ* = 0.154 nm) radiation under 40 kV, 40 mA and a scanning range of 10–80° 2θ. The morphological properties of the NPs were determined using transmission electron microscopy (TEM) with a JEOL JEM 1230 (Japan) operating at 120 KV. The surface analysis was carried out using X-ray photoelectron spectroscopy (XPS) ESCALAB250 xi XPS spectrometer with an Al K_α_ monochromatic source and a charge neutralizer. C 1 *s* peak at 284.5 eV are used as reference for binding energies. UV-Vis spectroscopy is used for determining the optical properties of the NPs using Agilent Cary 5000 UV–Vis spectrophotometer. N_2_ adsorption-desorption isotherms were measured at −195 °C using a model Gemini VII, ASAP 2020 automatic Micromeritics sorptometers (USA), equipped with an out gassing platform. Zeta potential (ζ) measurements were made using Zeta sizer Nano ZS, Malvern Instruments Ltd, Malvern (UK) for point of zero charge (pH_PZC_) determination.

### MB Photocatalytic experiments

The photocatalytic reactor is a Pyrex-glass cell with 1.0 L capacity. A 6W Lamp (BoittonInstruments) as the light source (365 nm) was placed in a quartz lamp holder which immersed in the photoreactor cell. Before illumination, the solution was allowed to stir in dark for 60 minutes to achieve adsorption–desorption equilibrium between the dye and photocatalyst. The cell was filled with 0.6 L of 10 mg/L of MB dye solution and 100 mg/L of the photocatalyst. The reactor was cooled down with an electric fan keeping the temperature at 25 °C. Magnetic stirrer was used to introduce fresh air bubbles into the suspension using a pump. MB degradation was examined by taking 4 mL of the suspension at 20 minutes irradiation time intervals. Finally, the rate of degradation was determined from the change in absorbance of MB solution.

### MB Adsorption experiments

The adsorption performance of the NPs was investigated by UV-Vis spectrometry. Typically, 100 mg/L of the NPs were added to a 10 mg/L of MB solution. The resultant suspensions were stirred in the dark for 120 min. Then, 3 mL of the solution was collected every at 30 minutes time interval.

## Results and Discussions

### Characterization of TiO_2_ NPs

**Figure 1:**
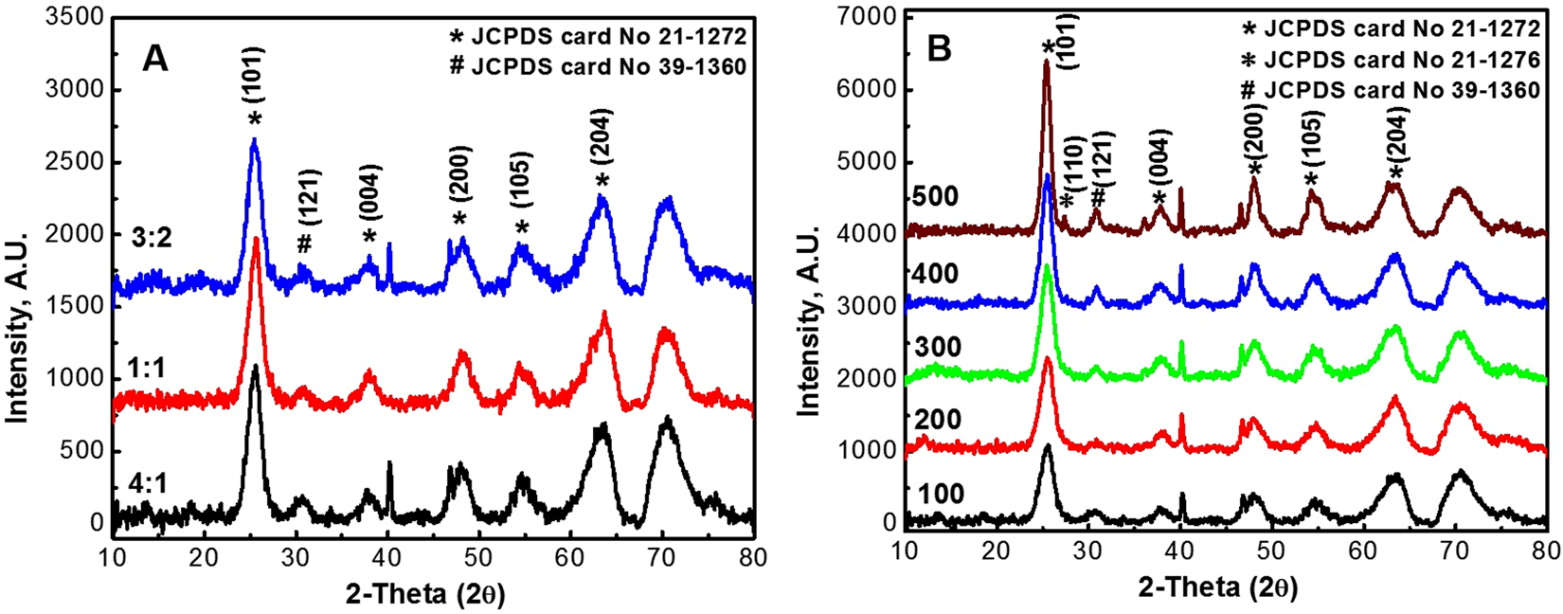
XRD patterns of TiO_2_ NPs at different (**A**) ethanol: water ratios (at 100 °C) and (**B**) temperatures (ratio 4:1 *v/v*).

The XRD patterns of the TiO_2_ NPs at various ethanol: water ratios and temperatures are shown in Fig. 1A,B. At a constant calcination temperature, ethanol: water ratios of 1:1, 4:1 and 3:2 (*v/v*) were investigated, Fig. 1A. The XRD patterns showed position of peaks consistent with the standard diffraction data for the different phases of TiO_2_ namely anatase and brookite^25^. For pure anatase (at JCPDS no. 21-1272) diffraction lines of 101, 004, 200, 105 and 204 were obtained. On the other hand, for pure brookite (JCPDS no. 39-1360), 121 diffraction line was observed. Consequently, based on these observations the resultant NPs had a mixed phase of anatase and brookite at the different ratios examined suggesting that the ratio has no influence on the NPs phase.

Figure 1B, demonstrates the effect of calcination temperatures on the prepared NPs at constant ethanol: water ratio (4:1 *v/v*). Patently, diffraction lines brookite and anatase manifested the spectra of temperatures between 100–400 °C. However, as the calcination temperature increased to 500 °C, an additional phase emerged resulting in a triple phase mixture (rutile, brookite and anatase). Thus, the calcination temperature had a significant effect on the NPs crystallinity compared to different solvent ratios. Therefore, TiO_2_ NPs prepared at 100 °C and 4:1 *v/v* ratio were used for further experiments.

**Figure 2:**
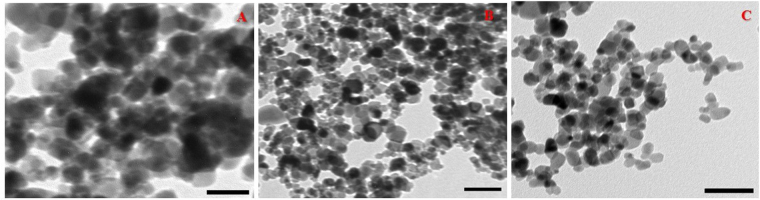
TEM of TiO_2_ NPs prepared at different pH values; (**A**) pH = 1.6, (**B**) pH = 7.0 and (**C**) pH = 10. The scale bar is 50 nm.

The Debye–Scherer equation was used to calculate the crystallite size of the TiO_2_ NPs prepared at 100 °C and 4:1 ethanol: water was found to be 4.5 nm. The effect of pH was explored on the NPs by adjusting the pH of the initial reaction solution (ethanol: water medium) at values of 1.6, 7.0 and 10. The morphological properties of the prepared NPs were studied using TEM, Fig. 2. All the NPs exhibited a semispherical morphology with particles size of 12.4 nm, 10.5 nm and 8.7 nm at pH values of 1.6, 7.0 and 10, respectively. Additionally, agglomeration of the NPs decreased as the pH of the solution increased, which can be ascribed to the Van der Waals attractive or electrostatic repulsion forces. Thus, we can assume that the attractive forces were dominant at pH 1.6 and 7.0. However, the repulsion forces outweighed the attractive forces at pH 10 resulting in less agglomerated particles^26^.

**Figure 3:**
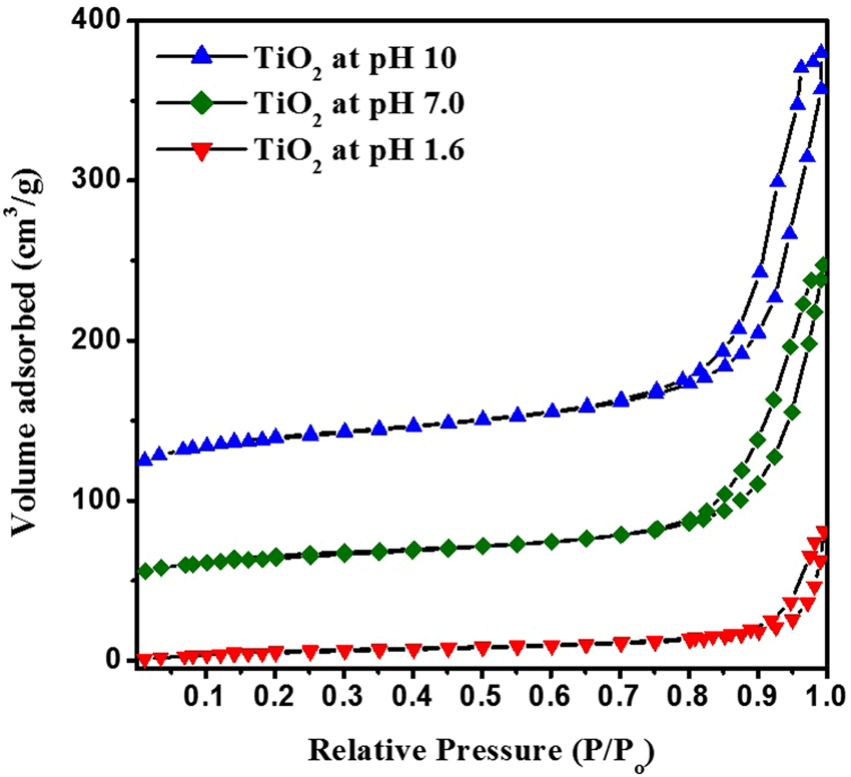
N_2_ adsorption-desorption isotherms of TiO_2_ NPs prepared at different pH values; 1.6, 7.0 and 10.

Figure 3 depicts the N_2_-isotherms of the prepared TiO_2_ NPs at different pH values. All NPs exhibited type-IV isotherm plots with different capillary condensation steps, which suggested the existence of mesopores in the prepared NPs.

The TiO_2_ BET surface areas were increased upon increasing the pH of preparation, this can be related to the increasing value of the pore volume. At P/P_o_ = 0.8–1.0, the hysteresis loop obtained of type H4 which can be ascribed to the narrow slit like pores due to the large inter aggregated pores enriched by the generation of hollow interiors^11^. Consequently, TiO_2_ NPs present a bimodal pore-size distribution ranging in the mesoporous region centered at 3.4 and >100 nm. Moreover, the capillary condensation arises at relatively high pressures and adsorption-desorption saturation is not significant due to the presence of large mesopores^27^. The measurements of N_2_-sorpometry confirmed the dependence of pore diameter, specific surface area, and the pore volume, for TiO_2_ NPs on the pH value as shown in Table 1.

**Table 1:** BET surface area (S_BET_), cumulative surface area (S_C_), average pore diameter (Dp) and volume (Vp) for the prepared NPs.

TiO_2_ NPs	*S*_*BET*_ (m^2^g^−1^)	*S*_*C*_ (m^2^g^−1^)	*D*p (nm)	*V*p (cm^3^g^−1^)
pH 10	183.6	215.7	10.8	0.50
pH 7.0	119.4	163.6	7.8	0.23
pH 1.6	12.4	12.7	8.4	0.03

**Figure 4:**
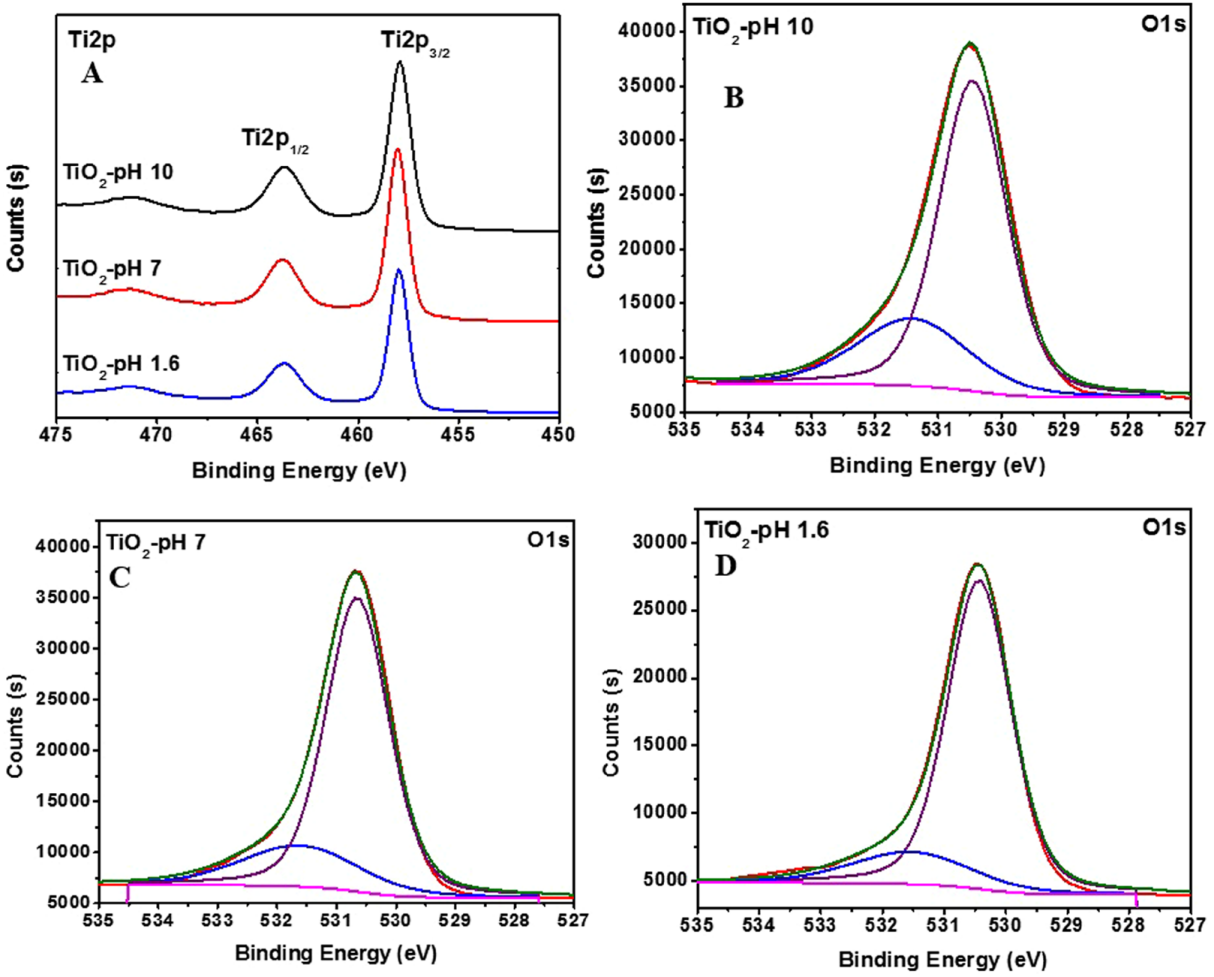
Deconvoluted XPS peaks for: (**A**) Ti 2 P, (**B**) O1s for pH 10, (**C**) O1s for pH 7.0 and (**D**) O1s for pH 1.6.

X-ray photoelectron spectroscopy was used to confirm the oxidation state and the surface composition of the synthesized TiO_2_ NPs at different pH as depicted in Fig. 4A–D. The binding energies obtained at 458.4 and 464.1 eV are assigned to Ti 2p_3/2_ and Ti 2p_1/2_, respectively^28^. The ratio of the areas of *A*(Ti 2p_1*/*2_)/*A*(Ti 2p_3*/*2_) was 0.5 and the difference in binding energy due to the spin orbital coupling, *ΔE*_b_ = *E*_b_(Ti 2p_1*/*2_) − *E*_b_(Ti 2p_3*/*2_) was 5.7 eV which is compatible with the reported and expected values^29^. The Ti 2p binding energies values did not changed while changing the pH. The O 1 s spectra (Fig. 4B–D) exhibited two peaks at binding energy of 530.4 eV and a small peak at 531.4 eV which are ascribed to O^2-^ species of the TiO_2_ and the surface hydroxyl (OH), respectively^30,31^. The effect of pH was observed on the hydroxyl oxygen peak. The percentages of hydroxyl oxygen were 11.1, 16.4 and 18.5% for pH values of 1.6, 7.0 and 10 respectively. The increase of hydroxyl oxygen percentage upon increasing the pH value is in agreement with previously published reports^32,33^.

**Figure 5:**
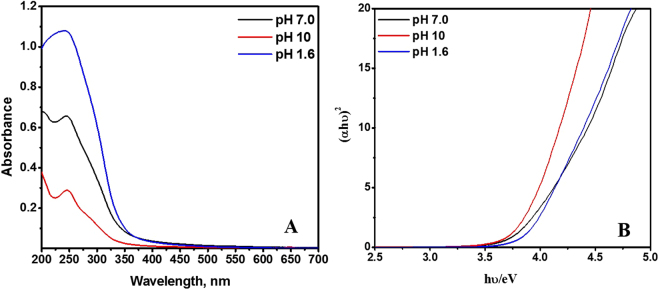
(**A**) UV-Vis absorption spectra obtained for TiO_2_ NPs prepared at different pH values; (**B**) Tauc plot for band gap determination.

Figure 5A displays the UV-Vis absorption spectra obtained for TiO_2_ NPs prepared under different pH conditions. All the NPs exhibited an absorbance at 350 nm, which is characteristic for TiO_2_ NPs. The band gap energies were calculated using the Tauc-equation:

1 $$({\boldsymbol{\alpha }}{\bf{h}}{\boldsymbol{\upsilon }})\,{\bf{1}}/{\bf{m}}={\bf{k}}\,({\bf{h}}{\boldsymbol{\upsilon }}-{{\bf{E}}}_{{\bf{g}}})$$

where E_g_ (the energy of optical band gap), k (constant) and m = 2 in the case of an indirect energy gap. (αhν)^2^ was plotted versus hν and the linear portion of the plot was extrapolated to the ordinate as shown in Fig. 5B. The results were found to be 3.54, 3.47 and 3.40 eV for TiO_2_ prepared at pH values of 1.6, 7.0 and 10, respectively, which are accepted for the TiO_2_ NPs.

### MB dye adsorption on the TiO_2_ NPs

**Figure 6:**
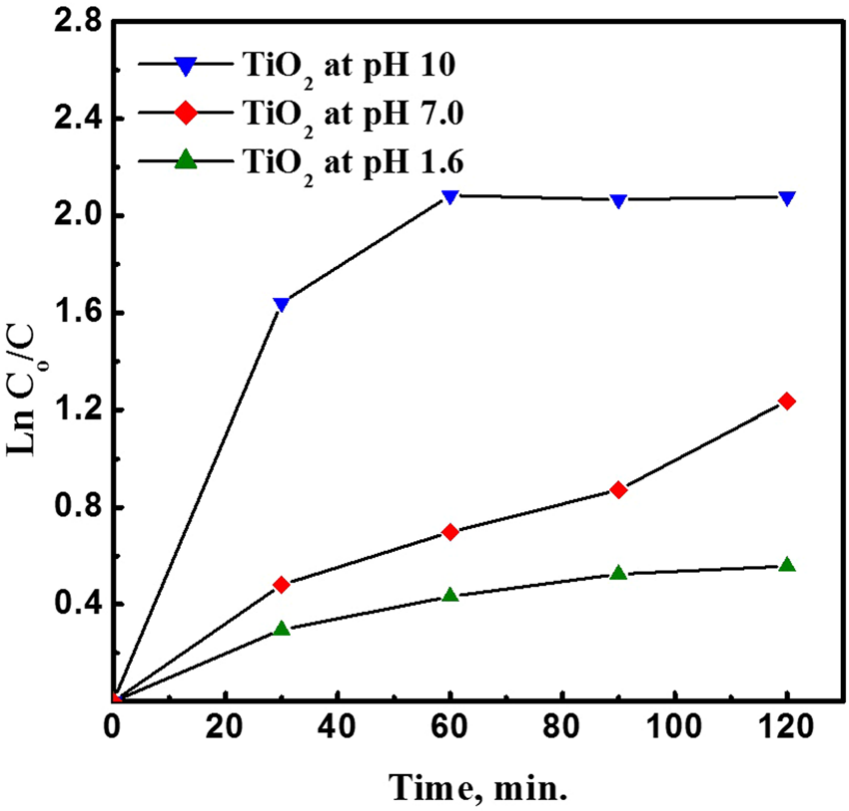
Adsorption kinetics of MB dye on TiO_2_ NPs surface prepared at different pH values; 1.6, 7.0 and 10.

The amount of organic pollutant adsorbed on the NPs surface is a crucial factor that influences their photocatalytic performance. Therefore, the adsorption of MB dye on the TiO_2_ NPs in the dark was monitored by measuring the absorbance values at different time intervals as shown in Fig. 6.

After 120 min without UV irradiation, the calculated rate constants were 0.0046, 0.010 and 0.017 min^−1^ for TiO_2_ NPs prepared at pH values of 1.6, 7 and 10, respectively. Furthermore, the adsorption percentages of MB on the NPs surface were 23.6, 71.0 and 87.5% for TiO_2_ NPs prepared at pH values of 1.6, 7 and 10, respectively. The variation in the adsorption percentage can be attributed to the nature of the electrostatic forces between MB dye and the surface of TiO_2_ NPs. In the case of the positively charged TiO_2_ (pH = 1.6) electrostatic repulsion was dominant between MB dye and the surface of TiO_2_ NPs which minimized MB adsorption. However, in the case of the negatively charged TiO_2_ NPs (pH = 7.0 and 10.0), electrostatic attraction promoted the adsorption of MB dye on their surface which was more preponderant at pH 10.

On the other hand, a control experiment was conducted in which MB dye was irradiated with UV light in the absence of the NPs. The findings showed that no major change in the MB dye concentration was observed and only 17% of the dye was degraded. This indicated that the direct photolysis of MB dye was insignificant in the absence of the NPs.

### Photocatalytic activity of TiO_2_ NPs

Determining the point of zero charge (pH_PZC_) is substantial to predict the charge on the NPs surface during the photodegradation process^34^. Since the photocatalysis occurs on the NPs surface, the performance of the photocatalyst is greatly influenced by the solution pH^35,36^, the pollutant type and the surface ability to adsorb the pollutant^37^.

**Figure 7:**
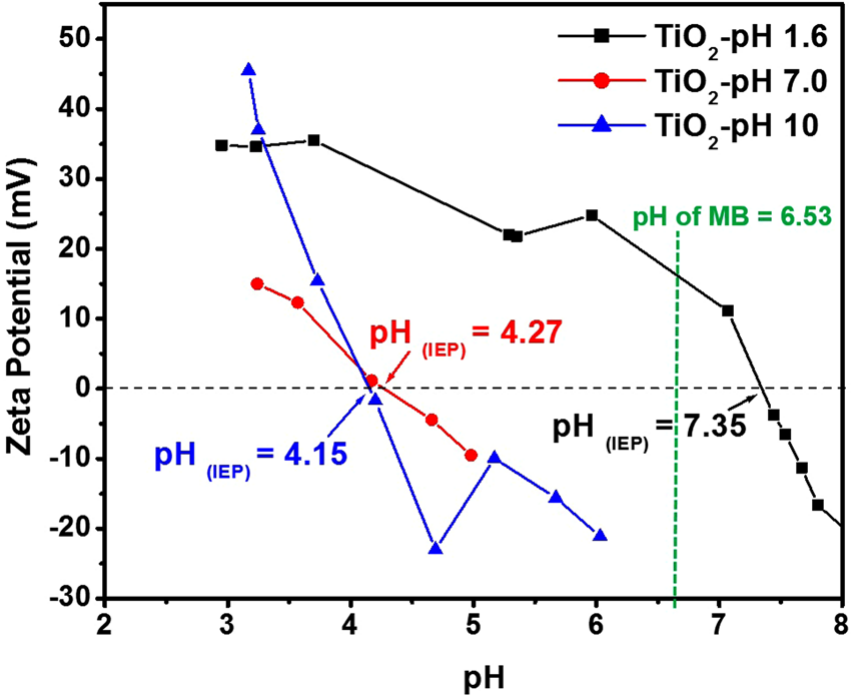
Zeta potential measurements of TiO_2_ NPs prepared at different pH values; 1.6, 7.0, and 10.

Figure 7 showed the Zeta potential of TiO_2_ NPs versus the pH of the solution and the pH of the MB was also indicated (green dotted line). The TiO_2_ NPs prepared at pH 1.6, 7.0 and 10 had pH_PZC_ of 7.35, 4.27 and 4.15, respectively. At pH values less than pH_PZC_ the NPs carried with a positive charge, whereas, higher pH values promote the formation of negative charge on the NPs.

**Figure 8:**
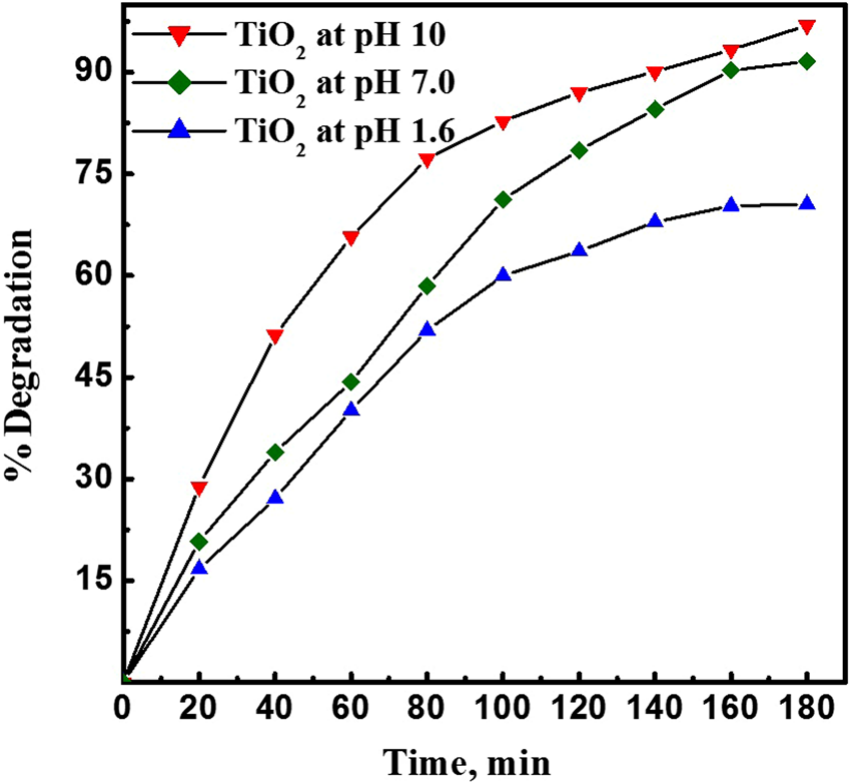
Photodegradation efficiencies of TiO_2_ NPs prepared at different pH values; 1.6, 7.0 and 10.

Photocatalytic degradations of MB (pH = 6.53) in an aqueous suspension of the TiO_2_ NPs prepared at different pH values were performed to evaluate their photocatalytic activity, Fig. 8 shows the degradation efficiency of the TiO_2_ NPs. The degradation efficiencies recorded were 73, 93 and 97% for TiO_2_ NPs prepared at pH values of 1.6, 7.0 and 10, respectively. The high efficiencies of TiO_2_ NPs prepared at pH 7.0 and 10 can be attributed to the presence of negative charge on their surfaces. Thus, the cationic dye MB with a positive charge can be adsorbed on the surface of the highly negatively charged TiO_2_ NPs through a strong electrostatic attraction and the electrostatic interaction was beneficial for enhancing the adsorptive property thereby enhancing the degradation efficiencies as in the cases of TiO_2_ NPs prepared at pH 7.0 and 10. On the other hand, TiO_2_ NPs prepared at pH 1.6 exhibited lower efficiency (73%) due to the electrostatic repulsion between the positively charged NPs and the cationic dye. These results are fully matched with the previously published reports^38^.

**Figure 9:**
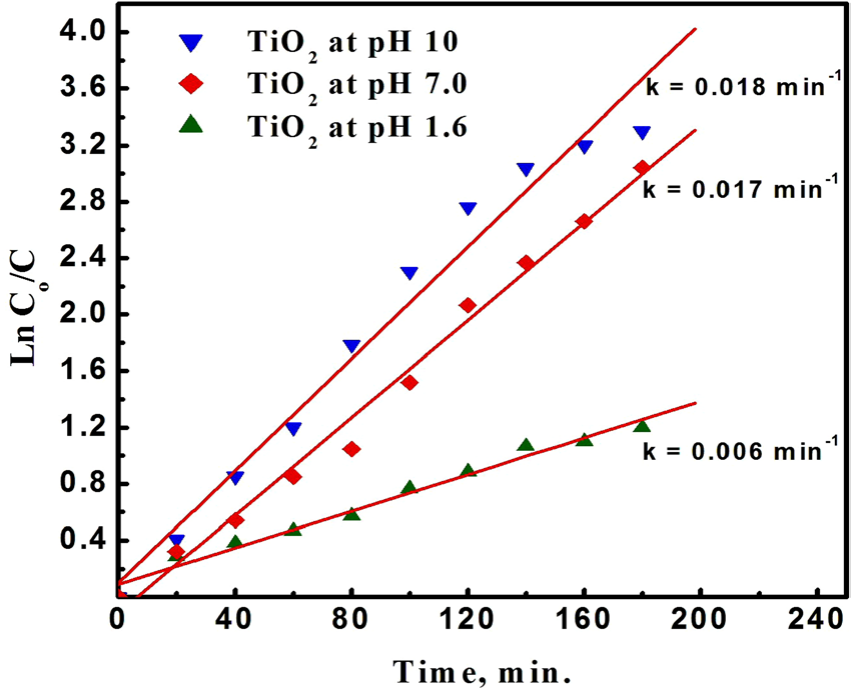
Pseudo first order rate kinetics for photocatalytic degradation of methylene blue dye using TiO_2_ NPs prepared at different pH values; 1.6, 7.0 and 10.

The photodegradation reaction follows a pseudo-first-order reaction. The photodegradation rate constant for the photodegradation reaction was determined from the equation:

2 $$\mathrm{ln}({{\rm{C}}}_{{\rm{o}}}/{\rm{C}})={\rm{kt}},$$

where C_o_ and C are the initial concentration and the concentration at time t, respectively, and k is the apparent first-order rate constant. A plot of ln C_o_/C versus time represents a straight line as shown in Fig. 9, where the slope of which upon linear regression equals the apparent first-order rate constant k.

The degradation rates obtained were 0.006 min^−1^, 0.017 min^−1^ and 0.018 min^−1^, for TiO_2_ NPs prepared at pH values of 1.6, 7.0 and 10, which were consistent with the results obtained from the photodegradation.

### Photodegradation mechanism

The photodegradation mechanism is based on the conversion of organic dyes into harmless gaseous CO_2_, nitrate, ammonium, and sulfate ions. The general photocatalysis degradation of the organic pollutant is given by the following scheme^36^:

1. Absorption of efficient photons
  3 $$({\rm{h}}{\rm{\nu }}\ge {\rm{Eg}}={\rm{3.2}}\,{\rm{eV}}\,{\rm{for}}\,{{\rm{TiO}}}_{{\rm{2}}}):{{\rm{TiO}}}_{{\rm{2}}}+{\rm{h}}{\rm{\nu }}\to {{\rm{e}}}_{{\rm{CB}}}^{-}+{{\rm{h}}}_{{\rm{VB}}}^{+}$$
2. Oxygen ionosorption:
  4 $${({{\rm{O}}}_{{\rm{2}}})}_{{\rm{ads}}}+{{\rm{e}}}_{{\rm{CB}}}^{-}\to {{\rm{O}}}_{{\rm{2}}}^{{\rm{\bullet }}-}$$
3. Neutralization of OH^−^ groups by photoholes to produce OH^•^ radicals
  5 $$({{\rm{H}}}_{{\rm{2}}}{\rm{O}}\iff {{\rm{H}}}^{+}+{{\rm{OH}}}^{-})\,{\rm{ads}}+{{\rm{h}}}_{{\rm{VB}}}^{+}\to {{\rm{H}}}^{+}+{{\rm{OH}}}^{{\rm{\bullet }}}$$
4. Neutralization of ${{\rm{O}}}_{2}^{-{\rm{\bullet }}}$ by protons
  6 $${{\rm{O}}}_{2}^{-{\rm{\bullet }}}+{{\rm{H}}}^{+}\to {{\rm{HO}}}_{2}^{{\rm{\bullet }}}$$
5. Transient hydrogen peroxide formation and dismutation of oxygen
  7 $${{{\rm{2HO}}}_{{\rm{2}}}}^{{\rm{\bullet }}}\to {{\rm{H}}}_{{\rm{2}}}{{\rm{O}}}_{{\rm{2}}}+{{\rm{O}}}_{{\rm{2}}}$$
6. Decomposition of H_2_O_2_ and second reduction of oxygen
  8 $${{\rm{H}}}_{{\rm{2}}}{{\rm{O}}}_{{\rm{2}}}+{{\rm{e}}}^{-}\to {{\rm{OH}}}^{{\rm{\bullet }}}+{{\rm{OH}}}^{-}$$
7. Oxidation of the organic reactant by OH• radicals
  9 $${\rm{R}}+{{\rm{OH}}}^{{\rm{\bullet }}}\to {{\rm{R}}}^{{\rm{\bullet }}}+{{\rm{H}}}_{{\rm{2}}}{\rm{O}}$$
8. Direct oxidation by reaction with holes

10 $${\rm{R}}+{{{\rm{h}}}^{+}}_{{\rm{VB}}}\to {{\rm{R}}}^{+{\rm{\bullet }}}\to {\rm{degradation}}\,{\rm{products}}$$

In this work, photo-holes are certainly not concerned by the initial step since the reactant is cationic and not electron donor. By contrast, the OH^•^ radicals can attack the R–S^+^=R functional group in MB, which is in direct coulombic interaction with titania’s surface as evidenced by the influence of the pH. Therefore, the initial step of MB degradation can be ascribed to the cleavage of the bonds of the R–S^+^=R functional group in MB into R–S(=O)–R, R–SO_2_–R, R–SO_3_H–R to finally produce SO_4_^2−^ and phenol^39^.

## Conclusion

A facile preparation of chargeable TiO_2_ NPs using modified hydrothermal method at different pH was reported. The synthesized NPs had small semispherical morphology with 12.4–8.7 nm in size and surface areas of 12.4–183.6 m^2^ g^−1^. Furthermore, the impact of surface charge of NPs on the photocatalytic activity was investigated via XPS to estimate the amount of hydroxyl groups and isoelectric measurements for pH_PZE_ determination Moreover, increasing the preparation pH value resulted in decreasing the particle size and increasing the surface area which played a significant role in addition to surface charge that govern the photo efficiency. The MB photodegradation results using different surface charges of TiO_2_ NPs influenced the degradation rate and the adsorption efficiency of the dye. The optimum efficiency obtained was 97% at a degradation rate of 0.018 min^−1^ using TiO_2_ NPs prepared at pH 10. This simple and robust approach can be applied to various types of nanophotcatalysts to manipulate their surface charge, hence enhancing their photocatalytic properties.
